# Hormone replacement treatment regimen is associated with a higher risk of hypertensive disorders of pregnancy in women undergoing frozen-thawed embryo transfer

**DOI:** 10.3389/fendo.2023.1133978

**Published:** 2023-02-24

**Authors:** Lijuan Fan, Na Li, Xitong Liu, Xiaofang Li, He Cai, Dan Pan, Ting Wang, Wenhao Shi, Pengfei Qu, Juanzi Shi

**Affiliations:** ^1^ Assisted Reproduction Center, Northwest Women’s and Children’s Hospital, Xi’an, China; ^2^ Translational Medicine Center, Northwest Women’s and Children’s Hospital, Xi’an, China

**Keywords:** frozen-thawed embryo transfer, endometrial preparation protocol, hormone replacement treatment, hypertensive disorders of pregnancy, obstetric outcomes

## Abstract

**Introduction:**

In frozen-thawed embryo transfer (FET) cycles, hormone replacement treatment (HRT) was associated with a higher risk of hypertensive disorders of pregnancy (HDP) compared with natural cycles (NC). Multiple pregnancy was a risk factor for HDP and several studies did not conduct subgroup analysis of singleton pregnancy and multiple pregnancy.

**Objective:**

To investigate whether HRT regimen could be a risk factor for HDP in women undergoing FET cycles in singleton and twin pregnancies.

**Methods:**

A retrospective cohort study at a tertiary hospital, including a total of 9120 women who underwent FET and achieved ongoing pregnancy; 7590 patients underwent HRT-FET and 1530 NC-FET. The main outcome was HDP. HDP were analyzed for singleton and twin pregnancies, respectively.

**Results:**

In the singleton pregnancy, the risk of HDP in the HRT-FET group was significantly higher than that in the NC-FET group (6.21% vs. 4.09%; *P*=0.003). After adjusting for female age oocyte pick up, female age at FET and body mass index (BMI), HRT was found as a risk factor for HDP (adjusted odds ration [aOR]: 1.43; 95% confidence interval [CI]: 1.07 to 1.91; *P*=0.017). In the multiple pregnancy, the risk of HDP in the HRT-FET and NC-FET groups was similar.

**Conclusion:**

HRT was associated with a higher risk of HDP in women who underwent FET and achieved singleton pregnancy.

## Introduction

The application of frozen-thawed embryo transfer (FET) has dramatically increased worldwide over the past decade for its merits. The FET enables the storage of excess embryos, reduces the incidence of ovarian hyperstimulation syndrome (OHSS), provides time required for preimplantation genetic testing (PGT), and facilitates fertility preservation. However, several studies have suggested that compared with fresh embryo transfer, the FET is associated with the reduced incidence rates of preterm birth, low birthweight (LBW), small for gestational age (SGA), and perinatal mortality (1). However, the safety of FET is challenged by the increased incidence rates of large for gestational age (LGA) and hypertensive disorders of pregnancy (HDP) (2).

The commonly used endometrial preparation regimens for FET include natural cycle (NC) and hormone replacement treatment (HRT) cycle. Given that the HRT cycles rely on exogenous hormone supplement and lack of corpus luteum, this condition might be less ‘physiological’ than a natural ovulatory cycle. Recent studies reported that HRT-FET were associated with a higher risk of HDP compared with NC-FET. However, several studies did not conduct subgroup analysis of singleton pregnancy and multiple pregnancy. Moreover, multiple pregnancy was reported as a risk factor for HDP, indicating its importance. Besides, the effects of the endometrium preparation method on the outcomes of pregnancies conceived with FET have not been fully clarified.

The present study aimed to assess the effects of different FET regimens on the risk of HDP in women who underwent FET cycles, and the risk of HDP was analyzed in singleton pregnancy and multiple pregnancy. This retrospective cohort study was conducted to compare the risk of HDP between NC-FET and HRT-FET groups.

## Materials and methods

### Study design and patients

This was a retrospective cohort study, in which the *in vitro* fertilization (IVF) was performed from January 2018 to December 2020 in the Center for Assisted Reproductive Technology of Northwest Women’s and Children’s Hospital (Xi’an, China). The protocol of the study was approved by the institutional review board of the hospital. Data were extracted from electronic medical records. Patients who underwent FET and achieved ongoing pregnancy were enrolled. Ongoing pregnancy was defined as the presence of at least one fetal heart pulsation on ultrasound beyond 20 weeks. All patients were enrolled only once. Women with chronic hypertension before pregnancy were excluded. Written informed consent was obtained from the participants before treatment.

### Controlled ovarian stimulation and vitrified cryopreservation

Ovarian stimulation protocols included gonadotropin-releasing hormone (GnRH) agonist protocol, GnRH antagonist protocol, and progestin-primed ovarian stimulation (PPOS) protocol. Recombinant human chorionic gonadotropin (OVIDREL; Merck Serono, Darmstadt, Germany) or GnRH-a (Decapeptyl; Ferring, Saint-Prex, Switzerland) were administered in patients when two leading follicles reached 18 mm in diameter. Oocyte retrieval was performed at 36 h after recombinant human chorionic gonadotropin or GnRH-a triggered by transvaginal ultrasound-guided aspiration. Insemination method was selected according to the sperm count after sperm preparation. A morphologic score of cleavage-stage embryo was given based on the number of blastomeres, the homogeneous degree of blastomeres, and the degree of cytoplasmic fragmentation, which has been extensively described in our previous study (3). If a couple has two or more high-quality cleavage-stage embryos on day 3 of embryo culture, the embryos were selected and cultured to blastocyst stage. Blastocyst evaluation was performed according to the Gardner’s grading system (4).

For patients who underwent GnRH agonist protocol and GnRH antagonist protocol, one to two fresh embryos were transferred into the uterus of women free of OHSS, hydrosalpinx, intrauterine adhesion and high progesterone level (> 1.5 ng/ml) on the day of triggering, and then, the spare embryos were cryopreserved for the next FET. Patients who underwent PPOS protocol had to freeze all their embryos. The vitrified cryopreservation was conducted according to standard protocols, as previously described (5).

### Endometrial preparation before FET

The selection of FET regimen is performed based on patients’ conditions, including menstrual regularity, ovulation regularity, doctors’ preference, endometrial development, and the prevalence of endometriosis and adenomyosis. For instance, patients with regular menstrual cycles and ovulation mainly undergo NC-FET. Patients with ovulation disorders or impaired endometrium development often undergo HRT-FET, because these patients have trouble in preparing the endometrium with natural ovulation. Meanwhile, HRT-FET is also selected due to the convenience of scheduling the date of FET. Patients with endometriosis, adenomyosis or recurrent implantation failure mainly undergo combination of GnRH-a and HRT-FET.

In this study, patients in the NC-FET group underwent transvaginal ultrasound on days 8 to 10 of the menstrual cycle. Follicular growth was monitored through transvaginal ultrasound and measurement of serum luteinizing hormone (LH). When the leading follicle had reached a mean diameter of >17 mm and the serum LH level was 20 IU/L, the transvaginal ultrasound was performed every day until ovulation. The day of ovulation was confirmed by transvaginal ultrasound. Cleavage-stage embryo and blastocyst-stage embryo were thawed and transferred on 3 and 5 days after ovulation, respectively.

For patients in the HRT-FET group, endometrial preparation was initiated with oral estradiol valerate (Progynova; Bayer, Berlin, Germany) at a daily dose of 4 mg from day 5 of menstrual cycle. For patients with impaired endometrial development, a daily maximum dose of 6 mg oral estradiol valerate and 3 mg transdermal 17-β estradiol (Besins Healthcare, Paris, France) were given. The serum progesterone level was measured and the transvaginal ultrasound was performed 10-12 days after the usage of exogenous estrogen. When the endometrial thickness reached 7 mm or more and the serum progesterone level was <1.5 ng/mL, exogenous progesterone was added. The FET was scheduled for 5 days for cleavage-stage embryos and for 7 days for blastocyst-stage embryos.

### Luteal support

Three methods of luteal support are implemented in our center. I. Vaginal progesterone gel (90 mg q.d; Crinone, Serono, Hertfordshire, UK); II. Vaginal progesterone soft capsules (0.2 g t.i.d; Utrogestan, Besins, France); III. Intramuscular progesterone (60 mg q.d; Xianju, Zhejiang, China). Patients from both groups could select one of these three luteal support methods and receive oral progesterone (10 mg t.i.d; Dydrogesterone, Abbott Biologicals B.V., Amsterdam, Netherlands) simultaneously. For patients who underwent HRT-FET, exogenous estrogen would be reduced after the confirmation of clinical pregnancy. The luteal support was maintained until week 10 of gestation.

### Definition of outcomes

The primary outcome was the risk of HDP. It was attempted to define HDP as sustained (on at least two occasions 6 h apart) blood pressure ≥ 140/90 mmHg after 20 weeks, with or without proteinuria and other signs or symptoms of preeclampsia and without a history of hypertension. As secondary outcomes, we analyzed some other perinatal risks and neonatal risks. Other perinatal risks included gestational diabetes mellitus (GDM), placenta previa, premature rupture of membrane, anemia, miscarriage, and stillbirth. Miscarriage was defined as the spontaneous loss of clinical pregnancy before 28 weeks of gestational age. Stillbirth was defined as the absence of signs of life at or after 28 weeks of gestation. Neonatal risks included preterm birth (<37 weeks’ gestation), extremely preterm birth (<32 weeks’ gestation), low birth weight (<2500 g), very low birth weight (<1500 g), and macrosomia (birth weight ≥4000 g) (6).

### Statistical analysis

Categorical variables were presented as count and proportion; normally distributed continuous variables were expressed as the mean and standard deviation, and abnormally distributed continuous variables were presented as the median and interquartile range (IQR). The Chi-squared test or the Fisher’s exact test were utilized to compare the categorical variables. The Student’s t-test was used to compare the continuous variables. The effects of different FET regimens on the risk of HDP and other outcomes were estimated using the generalized linear model adjusted for female age at oocyte pick up (OPU), female age at FET and body mass index (BMI). To assess the influence of potential heterogeneity on the risk of HDP, the effects of different FET regimens were estimated in several subgroups. All data were analyzed using the SPSS 22.0 software (IBM Corp., Armonk, NY, USA). The level of significance was set at *P* < 0.05.

## Results

### Baseline characteristics of patients

**Figure 1:**
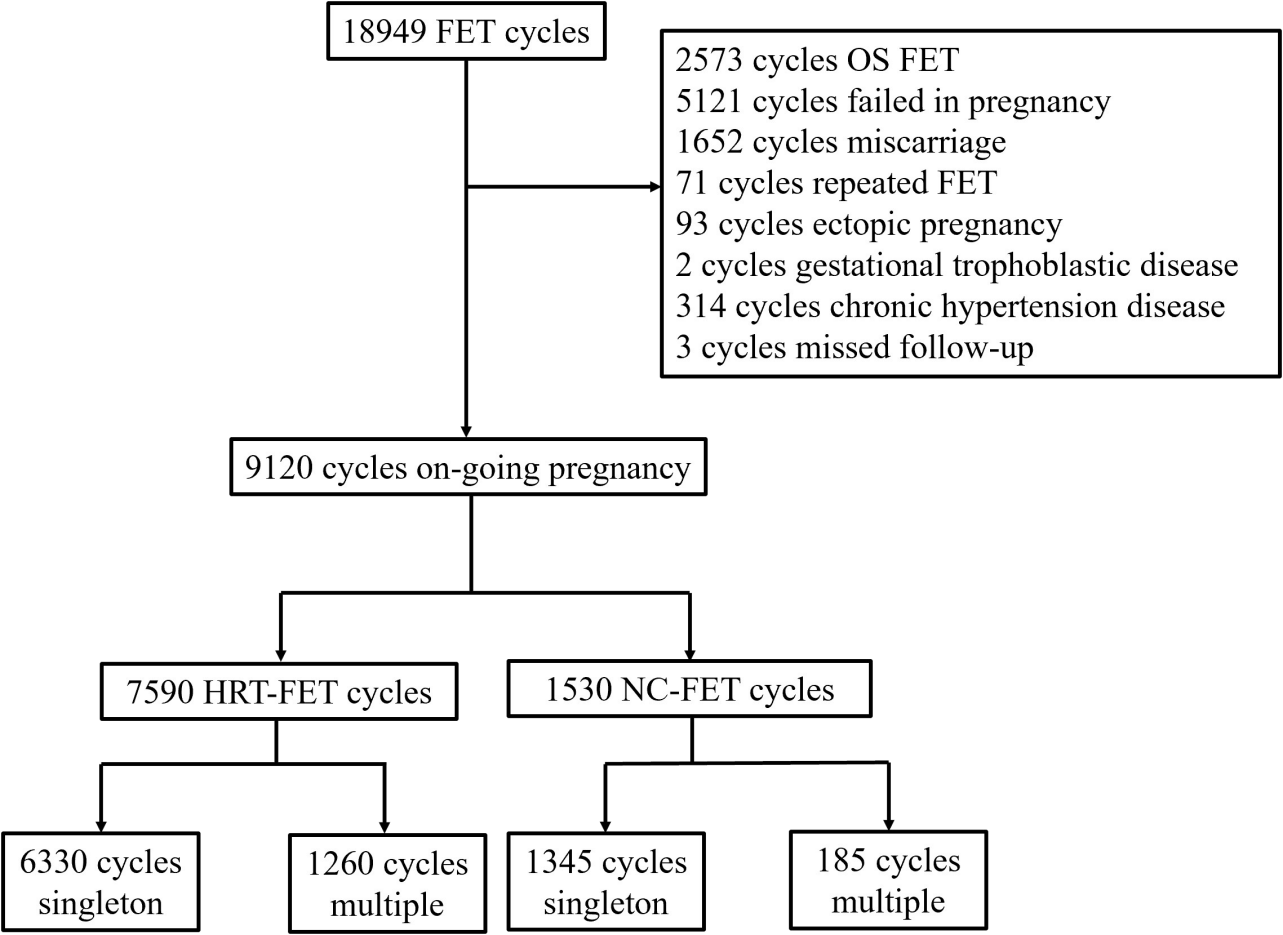
Patient selection flowchart.

A total of 9120 patients who fulfilled the inclusion and exclusion criteria were included in this study (**Figure 1**). Of these, 7590 and 1530 patients were in HRT-FET and NC-FET groups, respectively. The baseline characteristics of patients are shown in **Table 1**. Women who underwent HRT-FET were significantly younger at OPU (30.11 ± 3.90 vs. 30.40 ± 3.77, *P*<0.001) and FET (30.76 ± 3.91 vs. 31.37 ± 3.68, *P*<0.001) compared with those in the NC-FET group. Husbands of women in the HRT-FET group were significantly younger than those of women in the NC-FET group. Besides, a significantly higher BMI, a greater antral follicle count (AFC), a higher number of nulliparous women, and higher incidence rates of adenomyosis and endometriosis were detected in the HRT-FET group compared with the NC-FET group. After ovarian stimulation, the number of oocytes retrieved in the HRT-FET group was significantly greater than that in the NC-FET group. More patients in the HRT-FET group froze all their embryos compared with the NC-FET group. The endometrial thickness in the HRT-FET group was significantly thinner than that in the NC-FET group. In terms of the number of embryos transferred, it was higher in the HRT-FET group compared with that in the NC-FET group. The proportion of D3 cleavage-stage embryo transfer was significantly higher in the HRT-FET group compared with that in the NC-FET group. There were no significant differences in infertility duration, infertility type, proportion of patients with uterine cavity malformation, proportion of patients undergoing PGT, insemination type, and proportion of patients transferred at least one high-quality embryo between the two groups.

**Table 1:** Baseline characteristics of patients according to endometrium preparation protocols.

	NC-FET (n=1530)	HRT-FET (n=7590)	*P* value
Female age at OPU (y)	30.40 ± 3.77	30.11 ± 3.90	<0.001
Female age at FET (y)	31.37 ± 3.68	30.76 ± 3.91	<0.001
Male age (y)	32.84 ± 4.31	32.40 ± 4.64	<0.001
BMI, kg/m^2^	22.04 ± 3.10	22.53 ± 3.32	<0.001
Years of infertility	3.302 ± 2.32	3.41 ± 2.42	0.119
Antral follicle count (n)	11.92 ± 5.43	13.72 ± 6.60	<0.001
Type of infertility			0.503
Primary infertility, n (%)	827 (54.48)	4175 (55.42)	
Secondary infertility, n (%)	691 (45.52)	3359 (44.58)	
Parity			0.001
None, n (%)	1285 (84.71)	6548 (87.17)	
High order, n (%)	232 (15.29)	964 (12.83)	
Uterine cavity malformation, n (%)	42 (2.75)	167 (2.20)	0.082
Adenomyosis, n (%)	0 (0.00)	35 (0.46)	0.008
Endometriosis, n (%)	30 (1.96)	487 (6.42)	<0.001
PGT, n (%)	67 (4.38)	270 (3.56)	0.120
No. of oocytes retrieved (n)	11.98 ± 6.16	13.33 ± 6.93	<0.001
Insemination type			0.707
IVF, n (%)	1121 (73.65)	5595 (74.12)	
ICSI, n (%)	401 (26.35)	1954 (25.89)	
Freeze all, n (%)	914 (59.78)	5481 (72.21)	<0.001
Combined GnRH-a before FET, n (%)	0 (0.00)	3017 (39.75)	<0.001
Endometrial thickness (mm)	11.40 ± 1.90	10.42 ± 1.64	<0.001
No. of embryos transferred (n)	1.34 ± 0.48	1.47 ± 0.50	<0.001
Good quality embryo transfer			0.288
None, n (%)	355 (23.20)	1858 (24.48)	
≥1 high quality embryo, n (%)	1175 (76.80)	5732 (75.52)	
Type of embryo			<0.001
Cleavage-stage embryo, n (%)	279 (18.24)	1818 (23.95)	
Blastocyst-stage embryo, n (%)	1251 (81.77)	5772 (76.05)	

Continuous variables are presented as mean ± standard deviation, categorical variables are presented as number (percentage). *NC*, natural cycle; *FET*, frozen-thawed embryo transfer; *HRT*, hormone replace treatment; *OPU*, oocyte pick up; *BMI*, body mass index; *PGT*, pre-implantation genetic test; *IVF*, in vitro fertilization; *ICSI* intracytoplasmic sperm injection.

### Different FET regimens and the risk of HDP

The maternal and neonatal outcomes were categorized by the type of FET regimen. The risk of HDP was regarded as the primary outcome. Perinatal outcomes were analyzed in singleton pregnancy (**Table 2**) and twin pregnancy (**Table 3**), respectively. In the singleton pregnancy, the risk of HDP in the HRT-FET group was significantly higher than that in the NC-FET group (6.21% vs. 4.09%, *P*=0.003). After adjusting for confounders, including female age at OPU, female age at FET and BMI, the HRT-FET group was associated with a higher risk of HDP compared with the NC-FET group in singleton pregnancy (adjusted odds ratio (aOR): 1.43; 95% confidence interval (CI): 1.07 to 1.91; *P*=0.017). In the twin pregnancy, the risk of HDP was similar between the HRT-FET and NC-FET groups (10.48% vs. 6.49%, *P*=0.091).

**Table 2:** Perinatal outcomes of singleton pregnancy after two different FET regimens.

	NC-FET (n=1345)	HRT-FET (n=6330)	*P* value	Adjusted Model		
				OR/β	95%CI	*P* value
HDP, n (%)	55 (4.09)	393 (6.21)	0.003	1.43	1.07, 1.91	0.017
GDM, n (%)	98 (7.29)	421 (6.65)	0.399	0.86	0.68, 1.08	0.191
Placenta previa, n (%)	9 (0.67)	27 (0.43)	0.033	0.68	0.32, 1.47	0.327
Premature rupture of membranes, n (%)	7 (0.52)	67 (1.06)	0.067	1.87	0.86, 4.10	0.116
Anemia, n (%)	10 (0.74)	38 (0.60)	0.545	0.90	0.44, 1.84	0.780
Miscarriage, n (%)	38 (2.83)	230 (3.63)	0.143	1.25	0.88, 1.77	0.221
Still birth, n (%)	2 (0.15)	7 (0.12)	0.720	0.72	0.15, 3.56	0.690
Gestational age at birth (d)	272.85 ± 10.00	271.91 ± 12.58	0.011	–0.12	–0.228, –0.021	0.018
Method of delivery			<0.001			
Vaginal, n (%)	409 (31.29)	1312 (21.53)		1	Reference	
Cesarean section, n (%)	898 (68.71)	4781 (78.47)		1.70	1.49, 1.95	<0.001
Extremely preterm birth, n (%)	5 (0.38)	72 (1.18)	0.010	2.97	1.19, 7.39	0.019
Preterm birth, n (%)	85 (6.51)	586 (9.62)	<0.001	1.48	1.17, 1.88	0.001
Birth weight, kg	3.35 ± 0.49	3.35 ± 0.53	0.826	0.00	–0.04, 0.03	0.757
Macrosomia, n (%)	87 (6.66)	443 (7.27)	0.433	1.09	0.85, 1.38	0.505
Birth height (cm)	50.14 ± 1.47	50.00 ± 1.82	0.010	–0.13	–0.24, –0.03	0.013
Low birth weight, n (%)	48 (3.68)	309 (5.08)	0.033	1.35	0.99, 1.84	0.062
Very low birth weight, n (%)	4 (0.31)	47 (0.77)	0.065	2.31	0.83, 6.45	0.111

Continuous variables are presented as mean ± standard deviation, categorical variables are presented as number (percentage). *NC*, natural cycle; *FET*, frozen-thawed embryo transfer; *HRT*, hormone replace treatment; *HDP*, hypertensive disorders of pregnancy; *GDM*, gestational diabetes mellitus.

**Table 3:** Perinatal outcomes of multiple pregnancy after two different FET regimens.

	NC-FET (n=185)	HRT-FET (n=1260)	*P* value	Adjusted Model		
				OR	95%CI	*P* value
HDP, n (%)	12 (6.49)	132 (10.48)	0.091	1.60	0.86, 2.98	0.134
GDM, n (%)	11 (5.95)	59 (4.68)	0.455	0.77	0.40, 1.51	0.450
Placenta previa, n (%)	0 (0.00)	4 (0.32)	0.443	/	/	/
Premature rupture of membranes, n (%)	3 (1.62)	45 (3.57)	0.167	2.45	0.75, 8.02	0.140
Anemia, n (%)	6 (3.24)	9 (0.71)	0.002	0.19	0.06, 0.55	0.002
Miscarriage, n (%)	11 (5.95)	76 (6.03)	0.963	1.01	0.52, 1.96	0.978
Still birth, n (%)	0(0.00)	0(0.00)	/	/	/	/
Gestational age at birth (d)	253.80 ± 12.62	251.20 ± 14.69	0.027	–2.44	–4.74, –0.13	0.039
Method of delivery			0.456			
Vaginal, n (%)	9 (5.17)	47 (3.97)		1	Reference	
Cesarean section, n (%)	165 (94.83)	1137 (96.03)		1.20	0.57, 2.55	0.632
Extremely preterm birth, n (%)	7 (4.02)	64 (5.41)	0.444	1.31	0.58, 2.96	0.509
Preterm birth, n (%)	107 (61.495)	769 (64.95)	0.374	1.15	0.83, 1.60	0.401
Birth weight (kg)	2.52 ± 0.42	2.47 ± 0.48	0.044	2.76	1.11, 6.87	0.029
Macrosomia, n (%)	0	0	/	/	/	/
Birth height (cm)	48.02 ± 2.43	47.58 ± 3.06	0.011	–0.42	–0.76, –0.08	0.015
Low birth weight, n (%)	142 (40.81)	1071 (45.21)	0.123	1.20	0.95, 1.51	0.127
Very low birth weight, n (%)	5 (1.44)	94 (3.97)	0.019	2.76	1.11, 6.87	0.029

Continuous variables are presented as mean ± standard deviation, categorical variables are presented as number (percentage). *NC*, natural cycle; *FET*, frozen-thawed embryo transfer; *HRT*, hormone replace treatment; *HDP* hypertensive disorders of pregnancy; *GDM*, gestational diabetes mellitus.

### Different FET regimens and other perinatal outcomes

In the singleton pregnancy (**Table 2**), a higher cesarean section rate (78.47% vs. 68.71%, *P*<0.001) and a shorter gestational age at birth (271.91 ± 12.58 vs. 272.85 ± 10.00, *P*=0.011) were found in the HRT-FET group compared with the NC-FET group. This association remained essentially unchanged after adjusting for female age at oocyte retrieval, female age at FET and BMI (aOR: 1.70; 95% CI: 1.49 to 1.95; *P*<0.001) (aOR: −0.12; 95% CI: −0.23 to −0.02; *P*=0.018). A lower rate of placenta previa was detected in the NC-FET group compared with that in the HRT-FET group (0.63% vs. 0.67%), and this association was not significant after adjusting for covariates (aOR: 0.68; 95% CI: 0.32 to 1.47; *P*=0.327). Other perinatal outcomes, including the rates of GDM, premature rupture of membranes, anemia, miscarriage, and stillbirth were similar between the two groups in the singleton pregnancy (*P*≥0.05).

In the multiple pregnancy (**Table 3**), a lower rate of anemia (0.71% vs. 3.24%, *P*=0.002) and a shorter gestational age at birth (251.20 ± 14.69 vs. 253.80 ± 12.62, *P*=0.027) were identified in the HRT-FET group compared with those in the NC-FET group. This association remained essentially unchanged after adjusting for female age at oocyte retrieval, female age at FET and BMI (aOR: 0.19; 95% CI: 0.09 to 0.55; *P*=0.002) (aOR: −2.44; 95% CI: −4.74 to −0.13; *P*=0.039). Other perinatal outcomes, including the rates of GDM, placenta previa, premature rupture of membranes, miscarriage, stillbirth, and method of delivery were similar between the two groups in the multiple pregnancy (*P*≥0.05).

### Different FET regimens and neonatal outcomes

In the singleton pregnancy (**Table 2**), the rates of extremely preterm birth (1.18% vs. 0.38%, *P*=0.010) and preterm birth (9.62% vs. 6.51%, *P*<0.001) were significantly higher in the HRT-FET group than those in the NC-FET group. This association remained essentially unchanged after adjusting for female age at oocyte retrieval, female age at FET and BMI (aOR: 2.97; 95% CI: 1.19 to 7.39; *P*=0019) (aOR: 1.48; 95% CI: 1.17 to 1.88; *P*=0.001). Meanwhile, the birth height in the HRT-FET group (47.58 ± 3.06 cm) was significantly shorter than that in the NC-FET group (48.02 ± 2.43 cm, *P*=0.011). This association remained essentially unchanged after adjustment (aOR: −0.013; 95% CI: −0.24 to −0.03; *P*=0.013). Low birth weight rate was significantly higher in the HRT-FET group compared with that in the NC-FET group (5.08% vs. 3.68%, *P*=0.033), and this association was not significant after adjusting for female age at OPU, female age at FET and BMI (aOR: 1.35; 95% CI: 0.99 to 1.84; *P*=0.062). Other neonatal outcomes, including birth weight, macrosomia rate, and very low birth weight rate were similar between the two groups in the singleton pregnancy (*P*≥0.05).

In the multiple pregnancy (**Table 3**), the birth weight in the HRT-FET group was significantly lower (2.47 ± 0.48 vs. 2.52 ± 0.42 kg, *P*=0.044), and birth height (47.58 ± 3.06 vs. 48.02 ± 2.42 cm, *P*=0.011) was significantly shorter compared with the NC-FET group. The rate of very low birth weight (3.97% vs. 1.44% *P*=0.019) was significantly higher in the HRT group. These associations remained essentially unchanged after adjustment (aOR: 2.76; 95% CI: 1.11 to 6.87; *P*=0.029). There was no macrosomia in both groups. Other neonatal outcomes, including extremely preterm birth rate, preterm birth rate, birth weight, and low birth weight rate were similar between the two groups in the multiple pregnancy (*P*≥0.05).

### Subgroup analysis

The effects of two different FET regimens on the risk of HDP were analyzed in different subgroups (**Table 4**; **Supplementary Figure 1**). It was revealed that HRT-FET was associated with a higher risk of HDP in all the subgroups of women with different infertility durations, whether they attempted to freeze all their embryos or not and the number of embryos transferred, in which these associations were statistically significant (*P*<0.05). For the other subgroups, HRT-FET was associated with a higher risk of HDP compared with NC-FET. Meanwhile, those associations were statistically significant for the subgroups of female age at OPU (≤ 30, between 31 and 35), female age at FET (≤ 30, between 31 and 35), male age (≤ 35), female BMI (< 23 kg/m^2^), nulliparity, number of oocytes retrieved (< 10), insemination method (IVF), transfer of ≥1 high-quality embryo, and transfer of blastocyst-stage embryo (*P <*0.05).

**Table 4:** Subgroup analysis of different FET regimens on HDP.

	Subgroup	NC-FET	HRT-FET	OR	95%CI	*P* value
Female age at OPU (y)	≤30	629 (41.11)	3875 (51.05)	1.70	1.17, 2.47	0.005
	31–35	702 (45.88)	2819 (37.14)	1.72	1.12, 2.65	0.013
	≥36	199 (13.01)	896 (11.81)	1.16	0.58, 2.32	0.684
Female age at FET (y)	≤30	806 (52.68)	4438(58.47)	1.63	1.08, 2.47	0.020
	31–35	574 (37.52)	2431 (32.03)	1.91	1.27, 2.86	0.002
	≥36	150 (9.80)	721 (9.50)	1.08	0.58, 2.01	0.802
Male age (y)	<35	1251 (81.76)	6263(82.52)	1.83	1.33, 2.48	<0.001
	35–40	211(13.79)	959 (12.64)	1.10	0.64, 1.90	0.713
	>40	68 (4.44)	368(4.85)	1.74	0.57, 4.76	0.361
BMI (kg/m^2^)	<23	1031(67.39)	4651 (61.28)	1.53	1.07, 2.20	0.021
	23 –25	280 (18.30)	1398 (18.42)	1.80	0.95, 3.41	0.070
	>25	219 (14.31)	1541 (20.30)	1.30	0.81, 2.09	0.276
Infertility duration, year	≤3	959 (62.68)	4614 (60.79)	1.46	1.04, 2.05	0.029
	>3	571 (37.31)	2976 (39.21)	1.90	1.25, 2.87	0.003
Parity	None	1298 (84.84)	6626(87.30)	1.69	1.27, 2.25	<0.001
	High order	232 (15.16)	964 (12.70)	1.50	0.76, 2.97	0.246
No. of oocytes retrieved	<10	673 (44.51)	2831 (37.67)	1.89	1.25, 2.85	0.001
	10–14	446 (29.50)	2105 (28.01)	1.83	1.08, 3.11	0.155
	>14	393 (25.99)	2579 (34.32)	1.25	0.81, 1.94	0.218
Insemination type	IVF	1121 (73.65)	5595 (74.12)	1.90	1.37, 2.64	<0.001
	ICSI	401 (26.35)	1954 (25.88)	1.16	0.76, 1.79	0.489
Freeze all (%)	No	615 (40.22)	2109 (27.79)	2.04	1.31, 3.16	0.002
	Yes	914 (59.78)	5481 (72.21)	1.44	1.04, 1.99	0.027
Endometrial thickness (mm)	<9	673 (43.99)	2831 (37.30)	1.89	1.25, 2.85	<0.001
	9–12	464 (30.33)	2180 (28.72)	1.83	1.08, 3.11	0.155
	>12	393 (25.69)	2579 (33.98)	1.25	0.81, 1.94	0.218
No. of embryos transferred, n	1	1012 (66.14)	4063 (53.53)	1.53	1.11, 2.11	0.010
	≥2	518 (33.86)	3527 (46.47)	1.76	1.13, 2.74	0.013
Good quality embryo transfer	None	355 (23.20)	1858 (24.48)	1.30	0.79, 2.13	0.304
	≥1 high quality embryo	1175 (76.80)	5732 (75.52)	1.75	1.29, 2.38	<0.001
Type of embryo	Cleavage-stage	279 (18.24)	1818 (23.95)	1.74	0.92, 3.26	0.086
	Blastocyst-stage	1251 (81.76)	5772 (76.05)	1.61	1.21, 2.14	0.002

*OPU*, oocyte pick up; *FET*, frozen-thawed embryo transfer; *BMI* body mass index.

## Discussion

### Different FET regimens and the risk of HDP

Endometrial preparation protocols are commonly categorized into two categories: with and without corpus luteum protocols. HRT protocols combined with or without GnRH-a were classified into artificial preparation without corpus luteum, while with exogenous steroid. For patients with ovulation disorders, monitoring of ovarian follicular development is particularly troublesome. Preparing the endometrium with exogenous hormones is associated with some advantages, such as monitoring and scheduling of the timing of the procedure, making it more convenient and simpler. Therefore, HRT-FET cycle accounted for the majority of our FET cycles.

Hypertensive disorders were reported in 5.9% of assisted reproductive technology (ART) singleton and 12.6% of ART twin pregnancies (7). Multiple pregnancy was reported as a risk factor for HDP. Previous relevant studies have mainly compared the risk of HDP in NC and HRT groups without separate analysis of singleton pregnancy and multiple pregnancy. Therefore, we analyzed and reported the risk of HDP in singleton pregnancy and multiple pregnancy, respectively.

In our large retrospective cohort study of over 9120 FET cycles, the risk of HDP in the HRT-FET group was risen by 2.12% in the singleton pregnancy compared with that in the NC-FET group. In the multiple pregnancy, the risk of HDP in the HRT-FET group was escalated by 3.99% compared with that in the NC-FET group. This finding is similar to previously reported outcome (2, 8–10). Our results strengthened the pivotal etiopathogenic role of the corpus luteum. In HRT-FET cycles, the absence of corpus luteum was inevitably resulted in the lack of circulating vasoactive factors, such as relaxin (11), prorenin, and renin (12). These circulating vasoactive factors were required for maternal cardiovascular adaptation during the first trimester of pregnancy. Then, the lack of these circulating vasoactive factors may lead to the disruption of maternal circulatory adaptation. Von Versen-Höynck (11, 13) reported the decline of carotid-femoral pulse-wave velocity (cfPWV) and the increase of femoral pulse-wave transit time (fPWTT) during the first trimester in pregnant women who underwent HRT-FET compared with those who underwent NC-FET and fresh transfer.

Chen et al. (14) reported a significantly higher (two times) risk of preeclampsia in HRT-FET cycles compared with that in fresh transfer cycles in women who were diagnosed with polycystic ovary syndrome. The possible reason is that the supra-physiologic number of corpora lutea in fresh transfer cycles could produce the circulating vasoactive factors and protect pregnancies against HDP.

Another possible mechanism underlining the increased risk of HDP after HRT-FET may be attributed to the prematurely elevated level of estradiol. A low estradiol level during implantation allows for extravillous trophoblasts into uterine spiral arteries with vascular remodeling, and elevation of estradiol level later prevents further remodeling (15). In HRT-FET cycles, estradiol level is elevated more prematurely than that in NC-FET cycles. Trophoblastic invasion of spiral arteries may be suppressed by the prematurely elevated estradiol level (16). A retrospective study reported a lower risk of HDP in letrozole-induced FET cycles than that in HRT-FET cycles (17). The administration of letrozole during modified NC-FET cycles might potentially lower estradiol rise and optimal extravillous trophoblasts into uterine spiral arteries with vascular remodeling.

For China the average rate for cesarean section was 54.9 percent in 2014 (18). Higher rates of cesarean section were reported among women who get pregnant after ART. Meanwhile, patients present multiple pregnancy are prone to have greater need for cesarean section. These may be the reasons for the high rate of cesarean section in this study.

### Strengths and limitations

The major strength of this study is the large cohort size from a single-center, in which practice consistency can be assured. Controlled ovarian stimulation, IVF protocols, and laboratory conditions remained homogeneous. Additionally, maternal and neonatal outcomes of singleton and multiple pregnancies were analyzed separately, because multiple pregnancy was reported as a risk factor for HDP (19). Similar to multiple pregnancy, chronic hypertension, BMI>30 kg/m^2^, and female age were previously found as risk factors for HDP (20–22). We excluded women with chronic hypertension. Meanwhile, we adjusted female age at OPU, female age at FET and BMI in the assessment of the effects of different FET regimens on the risk of HDP. These analyses made our results more reliable.

Our study had several limitations. Firstly, this was a single-center retrospective study, in which inherent bias was inevitable. Regarding this deficiency, we screened patients with strict criteria.

Secondly, endometrial preparation was not randomly assigned in our study population, and imbalance in the number of enrolled patients in the two groups should be noted. However, ovulatory dysfunction was noted as the main indicator to pick HRT-FET rather than NC-FET. Ovulatory dysfunction was identified in approximately 15% of all infertile couples and accounted for up to 40% of female infertility (23). In our study, the number of patients in the HRT-FET group was 3.96 times greater than that in the NC-FET group. The main advantages of HRT-FET were its convenience, low-cost, and simplicity. Patients who underwent HRT-FET only need to visit the doctor for 2-3 times during their endometrial preparation period. Nevertheless, the imbalance in the number of study subjects could also be found in a previous study (24).

Thirdly, we failed in exact classification of patients into HDP categories after telephone follow-up. It is noteworthy that gestational hypertension and preeclampsia are involved in HDP (25). We followed up patients by telephone at one month after their expected date of delivery, thus, recall bias might be existed. Besides, some patients could not remember the exact diagnosis of HDP.

## Conclusion

In conclusion, HRT was found to be associated with a higher risk of HDP in women who underwent FET and achieved singleton pregnancy. However, further large-scale, prospective, randomized controlled trials with a longer follow-up are required to verify the increased risk of HDP in women undergoing HRT-FET.

## Data availability statement

The original contributions presented in the study are included in the article/**Supplementary Material**. Further inquiries can be directed to the corresponding author.

## Ethics statement

The studies involving human participants were reviewed and approved by Northwest Women’s and Children’s Hospital. The patients/participants provided their written informed consent to participate in this study.

## Author contributions

LF and NL: designed study, drafted the manuscript and reviewed the manuscript. XitL, XiaL, HC, DP, TW, WS and PQ: analyzed data. JS: Study conceptualization. All authors contributed to the article and approved the submitted version.

## Supplementary material

Supplementary Figure 1 Subgroup analysis of different FET regimens on HDP.
