# Supplementary material for: Hormone replacement treatment regimen is associated with a higher risk of hypertensive disorders of pregnancy in women undergoing frozen-thawed embryo transfer
Source: Front Endocrinol (Lausanne). 2023 Feb 24;14:1133978. doi: 10.3389/fendo.2023.1133978 (PMC9998903; doi:10.3389/fendo.2023.1133978)
Supplement: Supplementary Figure 1 — Subgroup analysis of different FET regimens on HDP. [file Image_1.pdf]

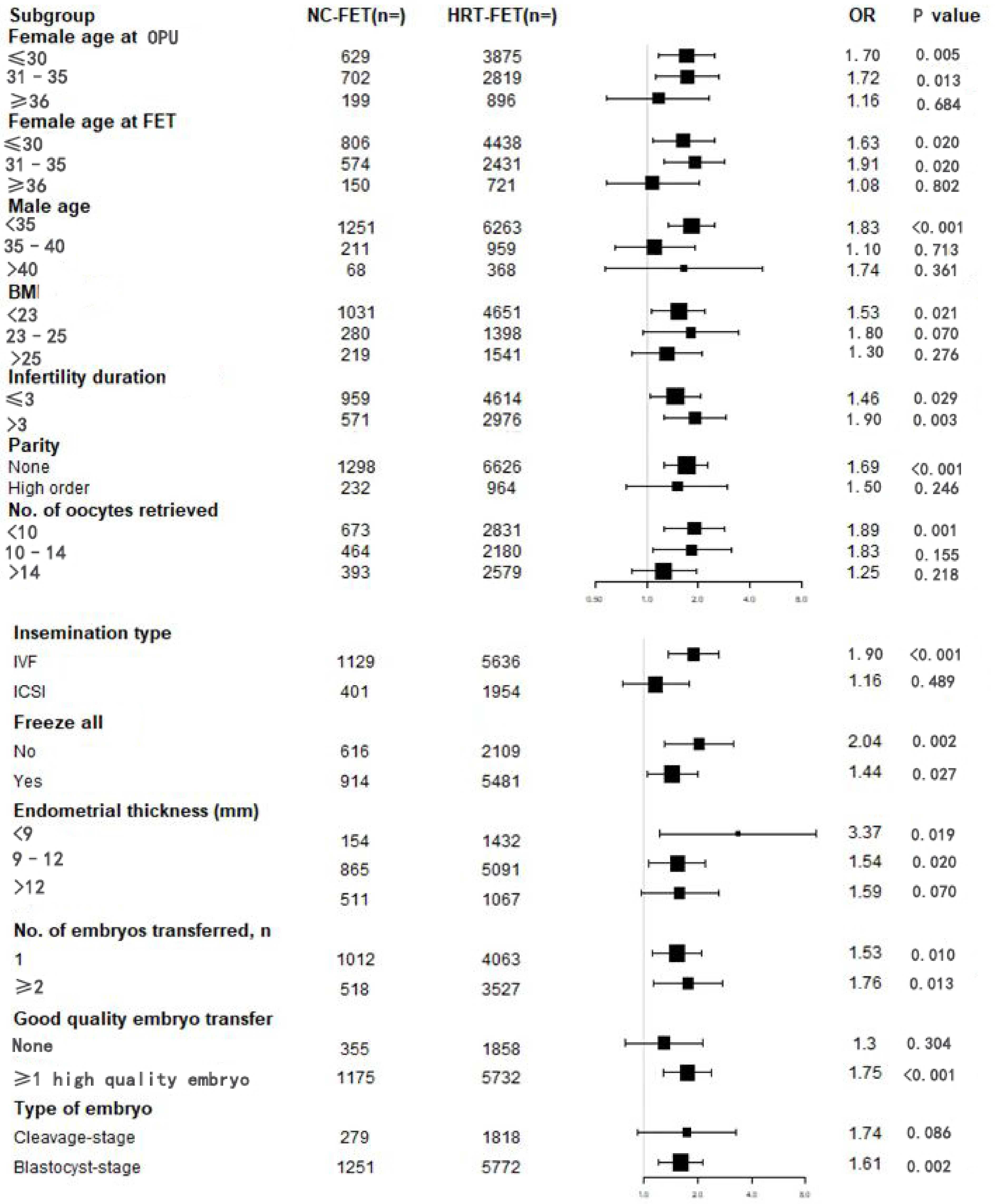
Subgroup NC-FET(n=) HRT-FET(n=) OR P value
Female age at OPU
⩽30 629 3875 1.70 0.005
31 - 35 702 2819 1.72 0.013
⩾36 199 896 1.16 0.684
Female age at FET
⩽30 806 4438 1.63 0.020
31 - 35 574 2431 1.91 0.020
⩾36 150 721 1.08 0.802
Male age
<35 1251 6263 1.83 <0.001
35 - 40 211 959 1.10 0.713
>40 68 368 1.74 0.361
BMI
<23 1031 4651 1.53 0.021
23 - 25 280 1398 1.80 0.070
>25 219 1541 1.30 0.276
Infertility duration
⩽3 959 4614 1.46 0.029
>3 571 2976 1.90 0.003
Parity
None 1298 6626 1.69 <0.001
High order 232 964 1.50 0.246
No. of oocytes retrieved
<10 673 2831 1.89 0.001
10 - 14 464 2180 1.83 0.155
>14 393 2579 1.25 0.218
Insemination type
IVF 1129 5636 1.90 <0.001
ICSI 401 1954 1.16 0.489
Freeze all
No 616 2109 2.04 0.002
Yes 914 5481 1.44 0.027
Endometrial thickness (mm)
<9 154 1432 3.37 0.019
9 - 12 865 5091 1.54 0.020
>12 511 1067 1.59 0.070
No. of embryos transferred, n
1 1012 4063 1.53 0.010
⩾2 518 3527 1.76 0.013
Good quality embryo transfer
None 355 1858 1.3 0.304
⩾1 high quality embryo 1175 5732 1.75 <0.001
Type of embryo
Cleavage-stage 279 1818 1.74 0.086
Blastocyst-stage 1251 5772 1.61 0.002
